# Effect of Short Photoperiod on Behavior and Brain Plasticity in Mice Differing in Predisposition to Catalepsy: The Role of BDNF and Serotonin System

**DOI:** 10.3390/ijms25052469

**Published:** 2024-02-20

**Authors:** Svetlana Adonina, Ekaterina Bazhenova, Darya Bazovkina

**Affiliations:** Federal Research Center Institute of Cytology and Genetics, Siberian Division of the Russian Academy of Science, Lavrentieva 10, Novosibirsk 630090, Russia; sveta-adonina@yandex.ru (S.A.); ekaterina.yu.bazhenova@gmail.com (E.B.)

**Keywords:** depressive-like behavior, catalepsy, short photoperiod, BDNF, brain serotonin, 5-HT receptors, HPLC, gene expression, mice

## Abstract

Seasonal affective disorder is characterized by depression during fall/winter as a result of shorter daylight. Catalepsy is a syndrome of some grave mental diseases. Both the neurotransmitter serotonin (5-HT) and brain-derived neurotrophic factor (BDNF) are involved in the pathophysiological mechanisms underlying catalepsy and depressive disorders. The aim was to compare the response of behavior and brain plasticity to photoperiod alterations in catalepsy-resistant C57BL/6J and catalepsy-prone CBA/Lac male mice. Mice of both strains were exposed for six weeks to standard-day (14 h light/10 h darkness) or short-day (4 h light/20 h darkness) conditions. Short photoperiod increased depressive-like behavior in both strains. Only treated CBA/Lac mice demonstrated increased cataleptic immobility, decreased brain 5-HT level, and the expression of *Tph2* gene encoding the key enzyme for 5-HT biosynthesis. Mice of both strains maintained under short-day conditions, compared to those under standard-day conditions, showed a region-specific decrease in the brain transcription of the *Htr1a*, *Htr4*, and *Htr7* genes. After a short photoperiod exposure, the mRNA levels of the BDNF-related genes were reduced in CBA/Lac mice and were increased in the C57BL/6J mice. Thus, the predisposition to catalepsy considerably influences the photoperiodic changes in neuroplasticity, wherein both C57BL/6J and CBA/Lac mice can serve as a powerful tool for investigating the link between seasons and mood.

## 1. Introduction

Seasonal affective disorder (SAD) is a mood disorder characterized by symptoms that occur usually during the darker, shorter days of fall and winter, with a remission the following spring or summer [1]. The symptoms of SAD generally include a carbohydrate craving, overeating, weight gain, depressed mood, drowsiness, and fatigue [2,3,4]. SAD is a considerable social problem: approximately 10% of the population in high or moderate latitudes suffer from depression in winter [4,5].

Neurotransmitter serotonin (5-HT) and brain-derived neurotrophic factor (BDNF) are principal players in the regulation of neuroplasticity and different kinds of behavior. It is important that BDNF interacts with the brain 5-HT system through feedback mechanisms [6]. The incredible polyfunctionality of 5-HT is mediated by 14 different 5-HT receptor subtypes expressed in mammals [7]. Among others, the 5-HT1A receptor has attracted particular attention because it is involved in the regulation of the functions of 5-HT neurons [8]. The body of data has demonstrated that the brain 5-HT is deeply involved in the pathophysiology of SAD [9,10]. In humans, seasonal variations have been reported for cerebrospinal fluid concentrations of 5-HT and its major metabolite, 5-hydroxyindoleacetic acid (5-HIAA), with the minimum levels found during the winter months [11]. Moreover, associations between SAD and *HTR2A* gene coding the 5-HT2A receptor [12] or SAD and 5-HTTLPR polymorphism in the 5-HT transporter promoter gene have been reported [13].

In its turn, BDNF is crucially involved in the regulation of neuro-, glio-, and synaptogenesis, neuroprotection [14,15], and in the response to stressful events [16,17]. The precursor of BDNF (proBDNF) is enzymatically cleaved, either by intracellular (furin/PC1) or extracellular proteases (tPA/plasmin/MMP), to generate mature BDNF [18]. Once secreted, BDNF and proBDNF bind to two sets of receptors. Whereas mature BDNF plays a crucial role in the neurogenesis promoting cell survival and differentiation via TrkB receptors, proBDNF as well as the other pro-neurotrophin precursor initiates apoptosis via the p75 receptor [15,19,20]. Data on BDNF and SAD interaction are scarce, however, it has been reported that serum BDNF concentrations show strong seasonal variation and correlations with the amount of ambient sunlight in persons with a DSM-IV depression diagnosis [21]. Moreover, seasonality and BDNF Val66Met polymorphism influences the depression outcome in patients [22].

Despite the large body of data on the participation of brain 5-HT and BDNF in short photoperiod-induced disorders, the molecular mechanisms underlying the pathogenesis of SAD remain not unclear. To solve this problem, it is possible to propose the use of animal models with known genetics, for example, laboratory mice of inbred strains. Because mice are nocturnal, it was believed that these animals are not photoperiod sensitive and would not be suitable models for SAD. However, mice of the C57BL/6 strain exposed for 4 weeks to short-day conditions showed an increased sucrose intake, increased depressive-like immobility in the forced swim test, and a decreased 5-HT level in some brain structures [23,24,25]. These changes are consistent with the current understanding of the symptoms and mechanism of SAD. Moreover, it has been demonstrated that the photoperiod may regulate the brain 5-HT system through similar mechanisms in diurnal or nocturnal animals [25].

Catalepsy, or freezing reaction, is a state of motor immobility; diseases such as Parkinson’s disease, schizophrenia, depression, and extrapyramidal disorders are often accompanied by extreme forms of catalepsy [26,27,28]. In some mice, so-called pinch catalepsy can be caused by a series of scruff pinches [29]. This non-drug catalepsy was not found in mice of the most common inbred strains, for example, BALB/c, C57BL/6J, DBA/2, or AKR/J. However, about 50% of CBA/Lac are predisposed to a pinch-induced freezing reaction [29]. It has been demonstrated that this hereditary catalepsy is associated with brain dysmorphology and altered stress response [30]. Moreover, high predisposition to catalepsy in mice has been shown to come with depressive-like features and sensitivity to BDNF and chronic SSRI (selective serotonin reuptake inhibitors) treatment [31,32]. It has been suggested that mice with a predisposition to catalepsy may be more sensitive to the effects of negative environmental factors such as a short photoperiod in comparison to catalepsy-resistant conspecifics.

The aim of the study was to compare the response of behavior and brain plasticity to photoperiod alterations in mice that genetically differed in predisposition to catalepsy. For these purposes, we intended to study (1) body mass, locomotor activity in the open field test, depressive like immobility in the forced swim test (FST), tail suspension test (TST), pinch-induced catalepsy test; (2) brain 5-HT system metabolism (in the hypothalamus, frontal cortex, hippocampus, and midbrain); (3) mRNA levels of genes encoding key elements of the brain 5-HT system (*Htr1a*, *Htr2a*, *Htr4*, *Htr7*, *Tph2*, *Scl6a4*) and mRNA levels of BDNF-related genes (*Bdnf*, *Ntrk2*, *Ngfr*, *Creb1*) in the brain structures (hypothalamus, frontal cortex, hippocampus, and midbrain) of catalepsy-resistant C57BL/6J and catalepsy-prone CBA/Lac mice exposed to standard and short-day conditions.

## 2. Results

### 2.1. Effects of Short Photoperiod on Body Weight

**Figure 1:**
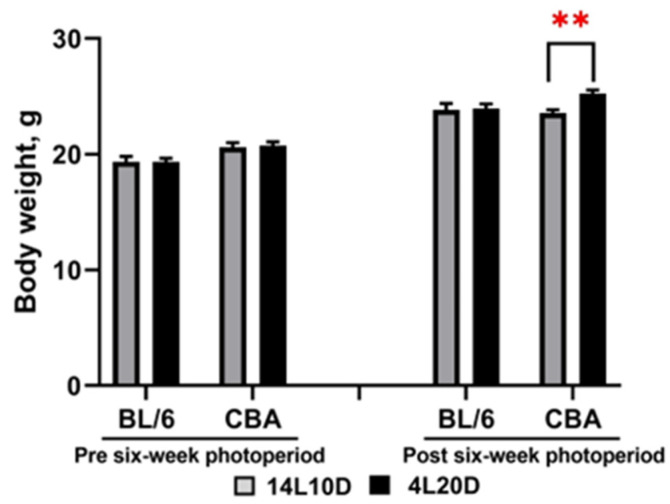
Photoperiodic changes in body weight (g) in catalepsy-resistant C7BL/6 (BL/6) and catalepsy-prone CBA/Lac (CBA) male mice. All mice were weighed before all experimental procedures (week 0) and before behavioral testing (week 6). ** *p* < 0.01 (red asterisks indicate the comparison to mice of the same strain under standard-day conditions), n = 10 mice per group.

The two-way ANOVA revealed the effect of the “genotype” factor (F(1,36) = 12.89, *p* < 0.001), but not of the “treatment” factor (F(1,36) < 1) and of their interaction (F(1,36) < 1) on body mass before all experimental procedures. Post hoc analysis did not find a difference between the 14L10D and 4L20D groups for BL/6 and CBA mice (*p* > 0.05). Therefore, males of each strain were divided into two weight balanced experimental groups (14L10D and 4L20D) (Figure 1). After 6 weeks of short photoperiod exposure, the two-way ANOVA revealed the effect of the “treatment” (F(1,36) = 5.03, *p* < 0.05) and of “genotype” × ”treatment” interaction at tendency (F(1,36) = 3.98, *p* = 0.053), but not of “genotype” factor (F(1,36) = 1.69, *p* > 0.05). Post hoc analysis revealed that a 6-week short photoperiod led to an increase in body weight only in CBA mice (*p* < 0.01) (Figure 1).

### 2.2. Effects of Short Photoperiod on Behavior

**Figure 2:**
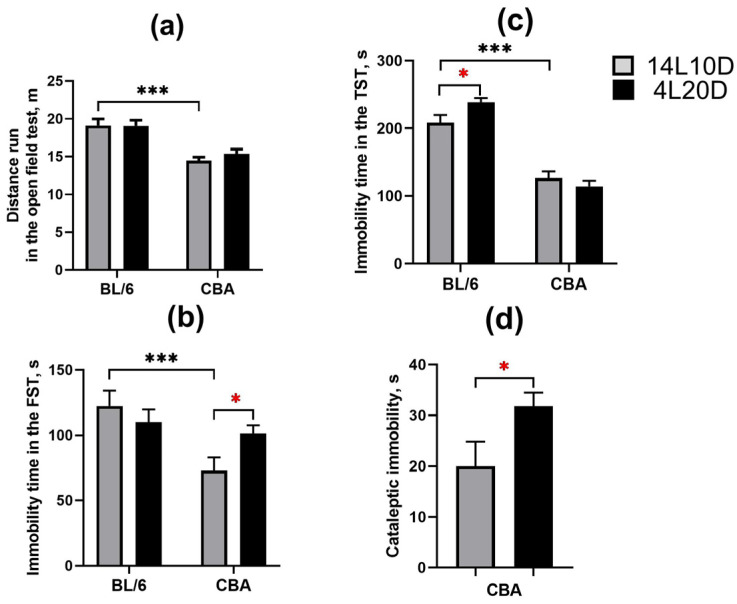
Short photoperiod effects on distance run in the open field test (**a**), immobility time in the forced swim test (**b**), immobility time in the tail suspension test (**c**), and immobility time in the pinch-induced catalepsy test (**d**) in catalepsy-resistant C7BL/6 (BL/6) and catalepsy-prone CBA/Lac (CBA) male mice. * *p* < 0.05, *** *p* < 0.001 (red asterisks indicate the comparison to mice of the same strain under standard-day conditions), n = 10 mice per group.

The two-way ANOVA revealed the effect of the “genotype” factor (F(1,36) = 39.04; *p* < 0.001), but not of the “treatment” factor (F(1,36) < 1) and of their interaction (F(1,36) < 1) on the distance run in the open field test. The short photoperiod did not affect locomotor activity in mice of both strains (*p* > 0.05) (Figure 2a).The two-way ANOVA demonstrated the effects of the “genotype” factor (F(1,36) = 9.08; *p* < 0.01) and of the “genotype” × ”treatment” interaction (F(1,36) = 4.56; *p* < 0.05) on the FST immobility time (no effect of the “treatment” factor (F(1,36) < 1) was observed). Exposure to the short photoperiod increased the FST immobility time only in the CBA mice (*p* < 0.05) (Figure 2b). The two-way ANOVA revealed the effects of the “genotype” factor (F(1,36) = 132.95; *p* < 0.001) and of the “genotype” × ”treatment” interaction (F(1,36) = 5.69; *p* < 0.05) on the TST immobility time (no effect of the “treatment” factor (F(1,36) < 1) was observed). Exposure to the short photoperiod increased the TST immobility time only in the BL/6 mice (*p* < 0.05) (Figure 2c). A significant effect of short photoperiod on the freezing time in the pinch-induced catalepsy test was found (F(1,18) = 4.64, *p* < 0.05). CBA mice exposed to short-day conditions demonstrated an increased cataleptic immobility duration compared to the control group (Figure 2d).

### 2.3. Effects of Short Photoperiod on Brain 5-HT Metabolism

Results of the two-way ANOVA analysis for the factors “genotype”, “treatment”, and their interaction on the level of 5-HT, its metabolite 5-HIAA, and the serotonin metabolic index (5-HIAA/5-HT) in the brain structures of BL/6 and CBA mice exposed to short photoperiod are presented in Table 1. 

**Figure 3:**
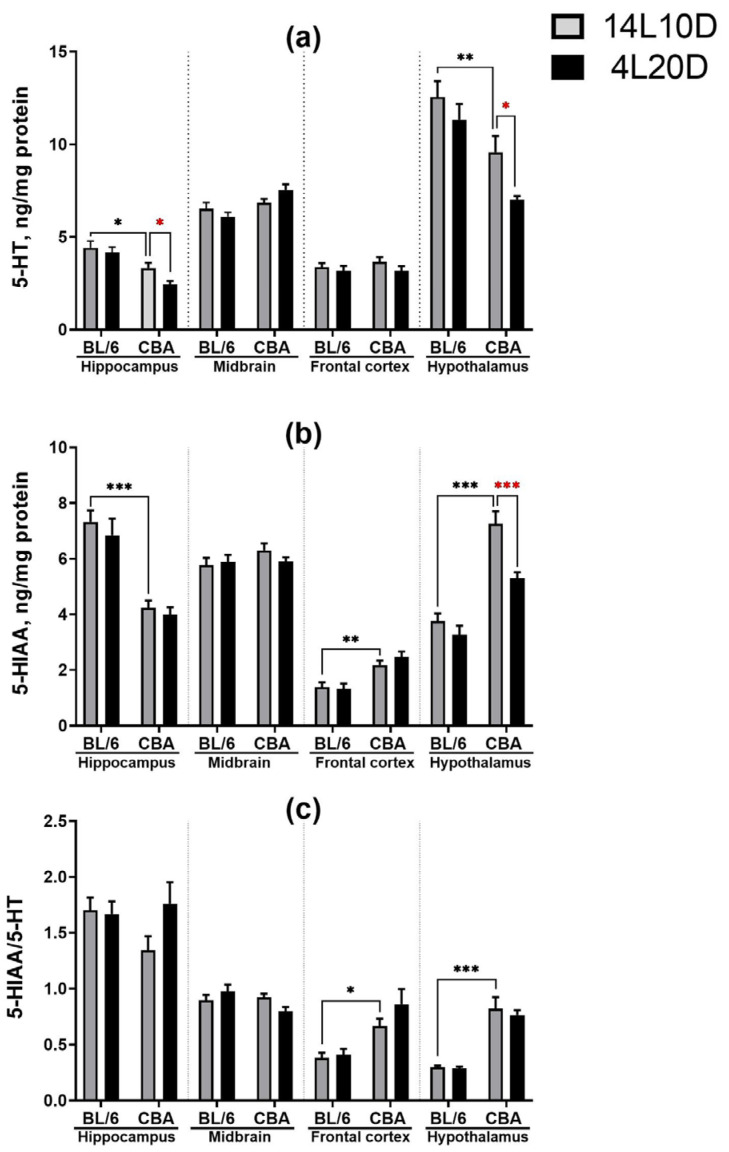
Short photoperiod effects on serotonin (5-HT) level (**a**), serotonin metabolite 5-HIAA level (**b**), and 5-HIAA/5-HT turnover index (**c**) in the brain structures of catalepsy-resistant C7BL/6 (BL/6) and catalepsy-prone CBA/Lac (CBA) male mice. * *p* < 0.05, ** *p* < 0.01, *** *p* < 0.001 (red asterisks indicate the comparison to mice of the same strain under standard-day conditions), n = at least 9 mice per group.

Post hoc analysis revealed that exposure to short-day conditions resulted in a reduced 5-HT level in the hippocampus (*p* < 0.05) and decreased 5-HT (*p* < 0.01) and 5-HIAA (*p* < 0.001) levels in the hypothalamus of CBA mice, but not of BL/6 animals (Figure 3a,b). However, the short photoperiod did not affect the 5-HIAA/5-HT ratio in mice of both strains (*p* > 0.05) (Figure 3c).

### 2.4. Effects of Short Photoperiod on mRNA Levels of 5-HT-Related Genes in the Brain

**Figure 4:**
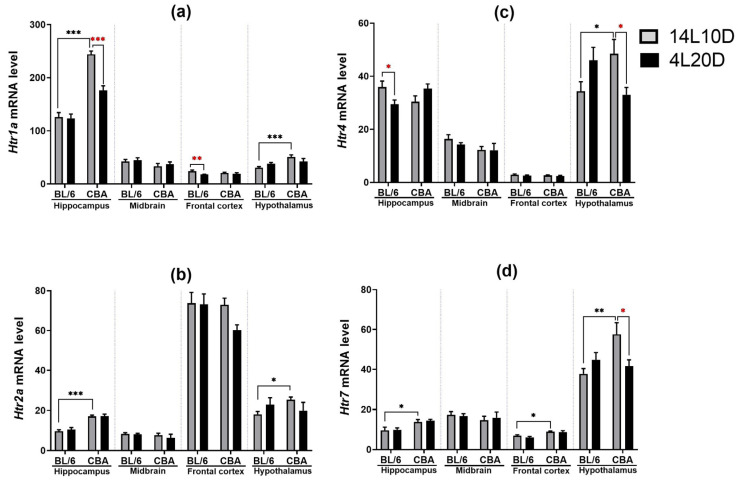
Short photoperiod effects on *Htr1a* (**a**), *Htr2a* (**b**), *Htr4* (**c**), and *Htr7* (**d**) mRNA level in the brain structures of catalepsy-resistant C7BL/6 (BL/6) and catalepsy-prone CBA/Lac (CBA) male mice. * *p* < 0.05, ** *p* < 0.01, *** *p* < 0.001 (red asterisks indicate the comparison to mice of the same strain under standard-day conditions), n = at least 8 mice per group.

Results of the two-way ANOVA analysis for the factors “genotype”, “treatment”, and their interaction on the mRNA levels of key genes of the 5-HT system in the brain structures of BL/6 and CBA mice exposed to a short photoperiod are presented in Table 2. Post hoc analysis revealed that only CBA mice maintained under short-day conditions demonstrated the reduced *Htr1a* mRNA level in the hippocampus of (*p* < 0.05) (Figure 4a). Post hoc analysis found no effect of the short photoperiod on the *Htr2a* mRNA level in the brain structures (Figure 4b), while a short photoperiod led to a reduction in the *Htr4* mRNA level in the hippocampus of BL/6 mice (*p* < 0.05) and hypothalamus of CBA mice (*p* < 0.05) (Figure 4c). Post hoc analysis revealed that exposure to short-day conditions induced the decrease in the *Htr7* mRNA level only in the hypothalamus of CBA mice (*p* < 0.05) (Figure 4d).

**Figure 5:**
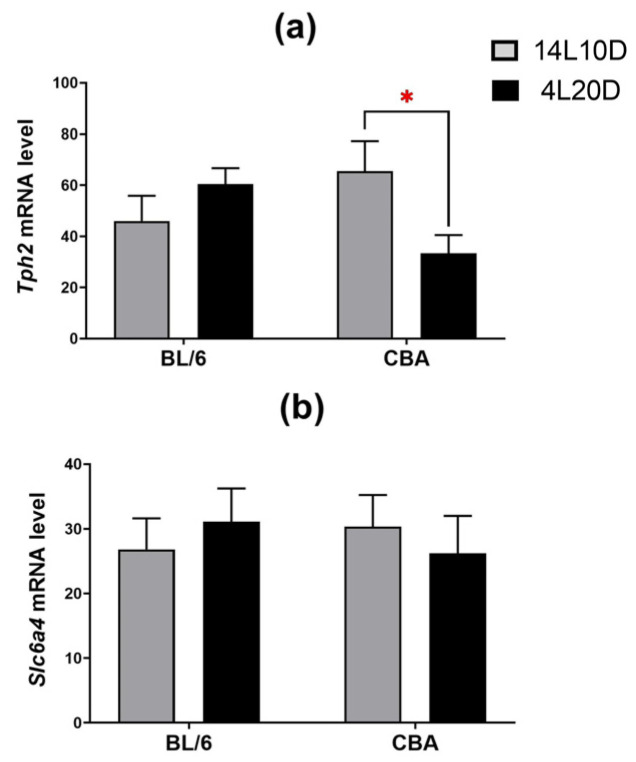
Short photoperiod effects on *Tph2* (**a**) and *Slc6a4* (**b**) mRNA levels in the midbrain of catalepsy-resistant C7BL/6 (BL/6) and catalepsy-prone CBA/Lac (CBA) male mice. * *p* < 0.05 (red asterisk indicates the comparison to mice of the same strain under standard-day conditions), n = at least 8 mice per group.

Post hoc analysis showed that exposure to short-day conditions resulted in a reduction in the *Tph2* mRNA level in the midbrain of CBA mice (*p* < 0.05), but not of BL/6 animals (Figure 5a). Post hoc analysis found no effect of short photoperiod on the *Slc6a4* mRNA level in the brain structures of both strains (*p* > 0.05) (Figure 5b).

### 2.5. Effects of Short Photoperiod on mRNA Levels of BDNF-Related Genes in the Brain

Results of the two-way ANOVA analysis for the factors “genotype”, “treatment”, and their interaction on the mRNA levels of BDNF-related genes in the brain structures of BL/6 and CBA mice exposed to a short photoperiod are presented in Table 3. 

**Figure 6:**
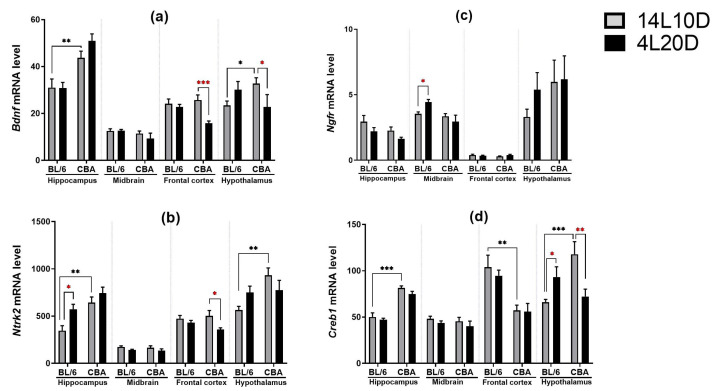
Short photoperiod effects on the *Bdnf* (**a**), *Ntrk2* (**b**), *Ngfr* (**c**), and *Creb1* (**d**) mRNA levels in the brain structures of catalepsy-resistant C7BL/6 (BL/6) and catalepsy-prone CBA/Lac (CBA) male mice. * *p* < 0.05, ** *p* < 0.01, *** *p* < 0.001 (red asterisks indicate the comparison to mice of the same strain under standard-day conditions), n = at least 8 mice per group.

Post hoc analysis revealed that exposure to short-day conditions resulted in a reduction in the *Bdnf* mRNA level in the frontal cortex (*p* < 0.001) and hypothalamus (*p* < 0.05) of CBA mice, but not of BL/6 animals (Figure 6a). Post hoc analysis showed a rise in the *Ngfr* mRNA level only in the midbrain of BL/6 animals exposed to a short photoperiod (*p* < 0.05) (Figure 6b). Post hoc analysis revealed that exposure to short-day conditions resulted in an increase in the *Ntrk2* mRNA level in the hippocampus (*p* < 0.05) and hypothalamus (*p* < 0.05) of BL/6 mice, but not of CBA animals as well as a decrease in the *Ntrk2* mRNA level only in the frontal cortex of CBA mice (*p* < 0.05) (Figure 6c). Post hoc analysis demonstrated that after a short photoperiod exposure, the *Creb1* mRNA level changed in different directions in the hypothalamus of mice of both strains: this was increased in BL/6 animals (*p* < 0.05) and decreased in CBA mice (*p* < 0.01) (Figure 6d).

## 3. Discussion

In the present study, we compared the response of behavior and brain plasticity to photoperiod alterations in catalepsy-resistant C7BL/6 (BL/6) and catalepsy-prone CBA/Lac (CBA) mice. A summary of the differences in the short photoperiod effects between both mouse strains is presented in Table 4.

To date, laboratory mice are actively used in the studies of photoperiodic effects on metabolism, brain function, and behavior. It has been shown that C57BL/6J mice under short-day exposure demonstrated higher [33] or lower body weights [34], lower glucose tolerance, and altered plasma metabolomic profiles compared to the mice under long-day conditions [23,33,34,35]. In addition, C57BL/6J mice exposed for a month to short-day conditions showed increased sucrose intake and depressive-like immobility in the forced swim test (FST) [23,24,35,36]. The results of the present work are consistent with these data. We demonstrated that a short photoperiod induced depressive-like behavior in the FST in CBA mice and in the tail suspension test (TST) in BL/6 mice.

The TST is likened to a “dry” version of the FST [37]. Despite the conceptual similarity between the TST and FST, there are subtle differences that are reflected in the findings in the assessment of antidepressant drugs [38]. It is likely that using both tests in the same study may provide a more reliable measure of depressive-like behavior. Discrepancies with other studies on FST results in BL/6 mice may be due to differences in the experimental design or conditions. For example, the experiments [24,36] were carried out on specific pathogen-free (SPF) conditions, which could significantly affect the behavioral results, since a short photoperiod may exert changes in immune processes [39].

Moreover, CBA mice exposed to the short-day condition weighed more in comparison to the control group. The fact that a short photoperiod had no effect on weight in the BL/6 animals is apparently due to the different experimental designs in our work and other studies. It should be noted that our results are in correlation with the symptoms observed in SAD patients such as overeating, weight gain, and depressed mood [2,3,4].

The phenomenon of pinch catalepsy is specific to the CBA/Lac mouse strain [29,30]. The selective breeding to high predisposition to catalepsy is associated with depressive-like features and sensitivity to BDNF and fluoxetine (selective 5-HT reuptake inhibitor) treatment [31,32]. It is noteworthy that the pathological manifestation of catalepsy (catatonic syndrome) occurs in affective disorders in patients [26,27]. In the present work, CBA mice demonstrated a significant increase in cataleptic immobility after prolonged exposure to short-day conditions. This result is in agreement with data obtained from the forced swim test, indicating the development of depressive-like behavior in CBA mice kept under a short photoperiod.

Many experimental and clinical studies have found a relationship between depressive disorders and serotonin deficiency in the brain [10,40]. Clinically effective antidepressants (for example, SSRI, selective serotonin reuptake inhibitors) increase the amount of serotonin available in the synapses [40,41]. We found that only CBA mice exposed to short-day conditions demonstrated decreased 5-HT and its metabolite 5-HIAA levels in the hypothalamus, a decreased 5-HT level in the hippocampus, and a reduced mRNA level of the *Tph2* gene encoding the key enzyme for 5-HT biosynthesis in the midbrain [10]. On the other hand, a short photoperiod did not affect the 5-HT metabolism in all of the studied brain structures of BL/6 mice. Our results are inconsistent with data that showed that C57BL/6 mice exposed to short-day conditions demonstrated a decreased 5-HT level in the amygdala [23,25] and midbrain [24,25], but not in the hypothalamus [23] and hippocampus [24]. These discrepancies may be caused by differences in the experimental designs and conditions. For example, the study in [24] was carried out at a SPF animal facility. It is known that short-day exposure can increase depressive-like responses through cytokine signaling [39], thus, the effects of a short photoperiod may be less in SPF mice. Additionally, the duration of the short-day condition could also be important. In [23], C57BL/6 mice were exposed to short-day conditions (8 h of light, 16 h of darkness) for 3 weeks before testing, while in present work, the short photoperiod conditions were more stringent: 4 h of light and 24 h of darkness for 6 weeks. This may be the reason for the different results in the photoperiodic effects on 5-HT metabolism in the hypothalamus.

The data on the short photoperiod effects on the expression of 5-HT receptor genes are extremely scarce. In the present work, mice of both strains showed a region-specific decrease in the mRNA of the *Htr1a*, *Htr4*, and *Htr7* genes in the brain structures, wherein most of the changes in the expression of these genes were found in the hypothalamus and hippocampus. On the one hand, there is no doubt that the 5-HT1A [42], 5-HT4 [43], and 5-HT7 receptors [44] play an important role in the pathogenesis and treatment of depressive disorders. On the other hand, many studies have indicated that hippocampal dysfunctions are involved in mood disorders via 5-HT mechanisms [45]. Our results are consistent with the data on altered hypothalamic 5-HT metabolism in SAD patients: it was shown that the 5-HT level in the postmortem hypothalamic tissue from human subjects was lower in the tissue collected in winter compared with the tissue collected in summer [46]. Given the role of hypothalamic 5-HT in feeding regulation, this could explain the characteristic of patients with SAD to desire carbohydrates and increased weight during winter depressive episodes [9].

Previous studies have shown the involvement of the 5-HT2A receptor in the treatment of affective disorders [7,47]. Moreover, associations between SAD and the *HTR2A* gene coding the 5-HT2A receptor have been observed [12]. To our surprise, no effect of a short photoperiod was found on the *Htr2a* mRNA level in all of the studied brain structures of mice of both strains. Meanwhile, the short-day exposure markedly increased *Htr2a* gene expression in the hippocampus and midbrain of B6-1473G mice with a genetically-induced reduction in TPH2 activity [24]. Taken together, these data suggest the important role of genotype in the short photoperiod effects on the brain 5-HT system. Thus, the increased depressive-like immobility observed in animals of both strains under short-day conditions was accompanied by a decrease in the functional activity of the 5-HT neurotransmitter system. Furthermore, these changes were more pronounced in the cataleptic CBA mice.

An important feature of inbred BL/6 and CBA mice is their difference in the pineal gland content of melatonin, a hormonal mediator of photoperiodic information [48]. Melatonin is synthesized from 5-HT and plays critical roles in the regulation of seasonality in mammals. However, most inbred mice including BL/6 (C7BL/6) are characterized by genetic defects in the melatonin biosynthesis pathway. In contrast, CBA mice produce this hormone [49,50]. Thus, different effects of a short photoperiod in both strains appear to be mediated by a melatonin secretion in CBA mice. Further investigations are needed to clarify how melatonin may contribute to the alterations of behavior and brain functions in melatonin-deficient and melatonin-proficient mice exposed to short-day conditions.

To our knowledge, this study is the first to examine the expression levels of genes related to the BDNF under short-day conditions: *Bdnf* encoding neurotrophin itself, *Ntrk2* encoding TrkB receptor (responsible for positive BDNF effects), *Ngfr* encoding p75 receptor (mediating a proapoptotic BDNF effects), and *Creb1* encoding CREB (cAMP-response element binding protein, transcription factor for this neurotrophin). Exposure to a short photoperiod had a significant region-specific effect on the BDNF-related genes in mice of both strains. The changes affected all brain structures, but the most impressive result was that the *Creb1* mRNA level in the hypothalamus was increased in BL/6 mice and decreased in those of CBA mice. The expression patterns of the *Bdnf* and *Ntrk2* genes in this structure in both strains of mice partially reproduced those of the *Creb1* gene. Our results are in agreement with data demonstrating the implication of the CREB/CRE transcriptional pathway in circadian clock timing in the suprachiasmatic nucleus, the locus of the master mammalian clock [51]. Furthermore, it was shown that the inhibition of hypothalamic CREB using a short-hairpin RNA lentiviral vector resulted in increased body-mass gain and reduced energy expenditure [52]. These findings are in agreement with the results of the present work, indicating a body weight rise in CBA mice exposed to a short photoperiod.

Accumulating evidence has demonstrated that the cross-talk between the BDNF and brain 5-HT system is involved in the development of behavioral disorders [6]. However, very few studies have investigated the role of BDNF–5-HT interaction in behavioral alterations induced by different daylight conditions. In chickens, constant light exposure in early life exhibited fear-related behaviors, which was associated with lower melatonin and 5-HT levels, a decreased expression of genes involved in the circadian clock, and BDNF in the hippocampus [53]. There is evidence that SSRI chronic treatment leads to increased *Bdnf* mRNA levels in the prefrontal cortex, hippocampus, and hypothalamus to augmented TrkB receptor and circadian rhythm gene expressions in the prefrontal cortex [54]. This finding is in agreement with the results of the present work, indicating brain 5-HT functional deficiency and a reduction in the mRNA level of BDNF-related genes in the frontal cortex and hypothalamus of CBA mice exposed to a short photoperiod.

Meanwhile, a short photoperiod produced the opposite effects on BDNF-related gene expression in the brains of BL/6 mice: we found a rise in the mRNA levels of *Creb1* in the hypothalamus, *Ntrk2* in the hippocampus and hypothalamus, and *Ngfr* in the midbrain. This significant difference from the CBA strain could possibly be due to the influence of the 5-HT system on BDNF-related gene expression in BL/6 mice. It was shown that 5-HT4 KO mice display depression-like behavior in the FST and sucrose consumption test [55]. Moreover, 5-HT4 knockout affected the *Bdnf* and *Ntrk2* mRNA levels in the mouse hippocampus [54] and desensitization of the 5-HT4 receptor increased CREB and BDNF protein expression in the rat hippocampus [56]. These data agree with our results, which revealed that the *Htr4* mRNA level was reduced in the hippocampus and augmented in the hypothalamus of BL/6 mice exposed to a short photoperiod. Moreover, it can be assumed that the increased expression of genes associated with BDNF may represent a compensatory mechanism that counteracts the negative effect of a short day on the 5-HT system of the brain. Thus, our results suggest the significant role of genotype in the short photoperiod effects on the BDNF system.

In conclusion, the predisposition to catalepsy considerably influences the photoperiodic changes in neuroplasticity markers. These alterations are likely to be controlled by genotype. Our study contributes to understanding the mechanisms underlying photoperiodic regulation of brain 5-HT and the BDNF, wherein both C57BL/6J and CBA/Lac mice can serve as a powerful tool for investigating the genetic link between seasons and mood.

## 4. Materials and Methods

### 4.1. Animals and Treatment

**Figure 7:**
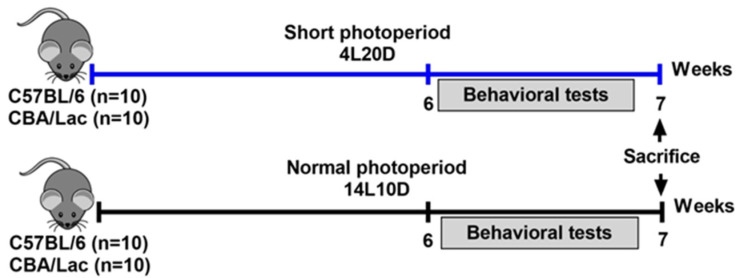
Experimental design performed in the study.

The experiments were carried out on male mice of catalepsy-resistant C57BL/6J (BL/6, N total = 20) and catalepsy-prone CBA/Lac (CBA, N total = 20) strains. After weaning, animals were kept in groups of five per cage (40 cm × 25 cm × 15 cm) under standard conditions (20–22 °C, free access to food and water, humidity 45–50%). The age of the animals was the same for all mice in the experiment. Male 6-week-old mice of both strains were exposed to standard conditions (14 h of light, 10 h of darkness, LD) or short-day conditions (4 h of light, 20 h of darkness, SD) for six weeks as described earlier (n = 10 animals per group) [24]. There were four experimental groups of 10 animals each: (1) BL/6 kept at 14L10D, (2) BL/6 kept at 4L20D, (3) CBA kept at 14L10D, and (4) CBA kept at 4L20D. All mice were weighed before all experimental procedures and before behavioral testing (after 6-week photoperiod exposure). Two days before the behavioral tests, the animals were isolated into individual cages to remove the group effect and kept at the same photoperiods during the tests. The sequence of behavioral tests was as follows: open field, forced swim test, tail suspension test (for all mice), and pinch-induced catalepsy test (only for CBA mice). Two days later, the animals were sacrificed via CO_2_ asphyxiation and then decapitated. The hypothalamus, frontal cortex, hippocampus, and midbrain were rapidly dissected, frozen in liquid N_2_, and stored at −80 °C. The brain regions were dissected by the same researcher based on a mouse brain atlas [57]. The following coordinates for the frontal cortex were used: AP: +1.6 to +2.8, L: −2 to +2; the thickness of the slice was about 1.5 mm. Hypothalamus was dissected using coordinates: AP: +0.3 to −2.9, L: −1 to +1, DV: 3.2–5.8. Both hippocampi were dissected from AP: −0.8 to AP: −2.9. For the midbrain, a cranial section was made in front of the superior colliculi (AP: −3) and a caudal section in front of the fossa rhomboidalis (AP: −7.3). All efforts were made to minimize the number of animals used and their suffering. The experimental design is shown in Figure 7.

### 4.2. Open Field Test

The open field test was carried out in an apparatus consisting of a round arena (40 cm in diameter, 30 cm wall height) with inverted illumination (two 12 W halogen lamps located 40 cm below it) and an EthoStudio computer registration system (Russia). The mouse was placed near the wall and the distance traveled (m) was recorded by the EthoStudio 2020 software for 5 min. The arena was carefully cleaned with wet and dry napkins after each test [58].

### 4.3. Forced Swim Test (FST)

The test was performed using a cylindrical glass tank (20 cm diameter, 30 cm height) filled with water (25 °C) for 60% of the volume. The tank was placed on a semitransparent platform with inverted illumination (two 12 W halogen lamps located 40 cm below it). The behavior was recorded during the 6 min test using EthoStudio 2020 software. The immobility time (s) was measured for the last 4 min of the test. The water was changed after each test [59,60].

### 4.4. Tail Suspension Test (TST)

The mouse was hung by the tail with a special adhesive tape to a device at the height of 40 cm. The behavior of the animal was recorded using EthoStudio 2020 software for 6 min. The level of depression-like behavior was assessed by the immobility time of the animal (s) [61].

### 4.5. Pinch-Induced Catalepsy

Catalepsy was tested according to a previously described procedure [29,30]. Animals were pinched with two fingers at the scruff of the neck and placed gently on parallel bars. The catalepsy duration was timed from the instant the mouse was released to the instant the animal moved its front paws from their original position or made movements of its body or head. A trial ended either when an animal started to move or after 2 min of freezing. Each animal was tested with 2-min intervals (the mouse was placed in its home cage between trials) until three positive responses were achieved, but no more than 10 times. Cataleptic time (s) was measured as the mean of three trials with the maximal values.

### 4.6. Tissue Preparation

The same brain samples were used for the total RNA extraction and evaluation of the serotonin (5-HT) and its metabolite (5-HIAA) levels. The brain samples were homogenized in 50 mM Tris HCl, pH 7.6, and 1 mM dithiotreitol using a motor-driven grinder (Z359971, Sigma-Aldrich, Darmstadt, Germany): hypothalamus and frontal cortex in 200 μL, hippocampus in 250 μL, midbrain in 350 μL. One aliquot of 50 μL of the homogenate was mixed with 150 μL of 0.6 M HClO_4_ for the 5-HT and 5-HIAA extractions (see Section 4.7). The rest of the homogenate was mixed with Trizol reagent (Bio Rad, Hercules, CA, USA) for the total RNA extraction (see Section 4.8).

### 4.7. Assay of 5-HT and 5-HIAA Levels

A total of 50 μL of brain tissue homogenate was mixed with 150 μL of 0.6 M HClO_4_ (see Section 4.6) and centrifuged for 15 min at 4 °C at 14,000 rpm to precipitate the protein. The supernatant was diluted with an equal volume of deionized water. The pellet was stored at −20 °C for a further protein concentration assay by the Bradford method [31]. The supernatant (20 μL) was analyzed using an HPLC system including an electrochemical detector (750 mV, DECADE IITM), glassy carbon VT03 flow cell (3 mm GC sb; Antec, Alphen a/d Rijn, The Netherlands), CBM-20A controller, LC-20AD pump, SIL-20A autosampler, and DGU-20A5R degasser (Shimadzu, Carlsbad, CA, USA). Chromatography was carried out in the isocratic mode at a flow rate of 0.6 mL/min on a Luna C18 column (length, 75 mm; inner diameter, 4.6 mm; particle size, 5 μm) with a C8 precolumn (Phenomenex, Torrance, CA, USA). The eluent was 10% methanol (Chimmed, Novosibirsk, Russia) in 50 mM KH2PO4 (Sigma-Aldrich, Burlington, MA, USA) containing 1.4 mM 1-octanesulfonic acid sodium salt (Chimmed, Novosibirsk, Russia) and 0.05 mM EDTA (Sigma-Aldrich, Burlington, MA, USA), pH 3.9 [35,36]. The calibration curves were plotted using serial dilutions containing 1, 2, and 3 ng of 5-HT and 5-HIAA. The peaks in the chromatograms were quantified with the LabSolution LG/GC program (Shimadzu, Carlsbad, CA, USA). The 5-HT and 5-HIAA contents were determined from the calibration curves and expressed in ng/mg protein, as assayed via the Bradford method and described elsewhere [23]. The index of 5-HT metabolism was evaluated as the 5-HIAA/5-HT ratio [60,61].

### 4.8. mRNA Level Assay by qPCR

The total mRNA was extracted from the homogenate and Trizol reagent mixture (see Section 4.6) and treated with RNAase-free DNAase (Promega, Madison, WI, USA) as recommended by the manufacturer. RNA concentration was determined spectrophotometrically with a Nanodrop 2000 (Waltham, MA, USA). Isolated RNA was diluted with water to the concentration of 0.12 μg/μL and stored at −80 °C. The presence of genomic DNA in the RNA preparations was assessed as described in [62,63]. Reverse transcription (RT) was performed using a random hexanucleotide primer and an R01 Kit as recommended by the manufacturer (Biolabmix, Novosibirsk, Russia). Synthesized cDNA was stored at −20 °C. The mRNA levels of the target genes were assayed via qPCR, using a set of selective primers (Table 5) and an R401 Kit (Sintol, Moscow, Russia) as recommended by the manufacturer (95 °C 5 min; (95 °C, 15 s; annealing temperature, 60 s; 82 °C, 2 s; fluorescence registration) × 40 cycles). Serial dilutions of genomic DNA with the concentrations of 0.125, 0.25, 0.5, 1, 2, 4, 8, 16, 32, and 64 ng/μL were amplified separately and used as external standards to plot the calibration curve in the Ct (number of threshold cycle)–lg P (logarithm of standard DNA quantity) coordinates using the LightCycler 480 software (release 1.5.1). To verify the specificity of amplification, the melting curves of the qPCR products were obtained and analyzed for each reaction and each primer pair. The gene expression was presented as a ratio of the number of cDNA copies to 100 copies of *Polr2a* cDNA used as an internal standard [62,63].

### 4.9. Statistics

All values were presented as means ± SEM and compared with two-way factorial ANOVA (factors “genotype” and “treatment” as well as their interaction) followed by Fisher’s post hoc analysis when appropriate. The data from the catalepsy test were processed using one-way analysis of variance (ANOVA). The statistical significance was set at *p* < 0.05. Data distribution was controlled for normality using the Kolmogorov–Smirnov and Shapiro–Wilk tests. The Dixon test was used to find and exclude outliers from the analysis.

## Figures and Tables

**Table 1:** ANOVA results for the effects of genotype, photoperiod, and their interaction on serotonin (5-HT), its main metabolite 5-hydroxyindoleacetic acid (5-HIAA), and 5-HIAA/5-HT ratio in the different brain areas of catalepsy-resistant C57Bl/6 and catalepsy-prone CBA/Lac mice.

	Effect of Genotype	Effect of Photoperiod	Effect of Interaction
5-HT			
Hippocampus	F(1,36) = 22.81 ***	F(1,36) = 3.97, *p* = 0.054	F(1,36) = 1.11, *p* > 0.05
Midbrain	F(1,36) = 10.12 **	F(1,36) < 1	F(1,36) = 4.01, *p* = 0.053
Frontal cortex	F (1,35) < 1	F(1,35) = 1.93, *p* > 0.05	F(1,35) < 1
Hypothalamus	F (1,35) = 24.30 ***	F(1,35) = 6.52 *	F(1,35) < 1
5-HIAA			
Hippocampus	F(1,36) = 53.49 ***	F(1,36) < 1	F(1,36) < 1
Midbrain	F(1,36) = 1.37, *p* > 0.05	F(1,36) < 1	F(1,36) = 1.20, *p* > 0.05
Frontal cortex	F(1,35) = 28.50 ***	F(1,35) < 1	F(1,35) < 1
Hypothalamus	F(1,35) = 68.02 ***	F(1,35) = 13.21 ***	F(1,35) = 4.74 *
5-HIAA/5-HT			
Hippocampus	F(1,36) < 1	F(1,36) = 1.83, *p* > 0.05	F(1,36) = 2.57, *p* > 0.05
Midbrain	F(1,36) = 2.83, *p* > 0.05	F(1,36) < 1	F(1,36) = 4.99 *
Frontal cortex	F(1,35) = 19.03 ***	F(1,35) = 1.74, *p* > 0.05	F(1,35) < 1
Hypothalamus	F(1,35) = 72.89 ***	F(1,35) < 1	F(1,35) < 1

Genotype: C57Bl/6 (BL/6) or CBA/Lac (CBA). Photoperiod: standard (14L10D) day or short (4L20D) day. Interaction: genotype × photoperiod. * *p* < 0.05; ** *p* < 0.01; *** *p* < 0.001 (significant values are shown in bold).

**Table 2:** ANOVA results for the effects of genotype, photoperiod, and their interaction on the mRNA level of key genes in the serotonin system in different brain areas of catalepsy-resistant C57Bl/6 and catalepsy-prone CBA/Lac mice.

	Effect of Genotype	Effect of Photoperiod	Effect of Interaction
*Htr1a*			
Hippocampus	F(1,35) = 116.43 ***	F(1,35) = 19.56 ***	F(1,35) = 17.05 ***
Midbrain	F(1,34) = 3.61, *p* = 0.066	F(1,34) < 1	F(1,34) < 1
Frontal cortex	F(1,36) < 1	F(1,36) = 5.88 *	F(1,36) = 2.79, *p* > 0.05
Hypothalamus	F(1,33) = 6.11 *	F(1,33) < 1	F(1,33) = 4.71 *
*Htr2a*			
Hippocampus	F(1,35) = 64.24 ***	F(1,35) < 1	F(1,35) < 1
Midbrain	F(1,34) = 1.31, *p* > 0.05	F(1,34) < 1	F(1,34) < 1
Frontal cortex	F(1,36) = 2.36, *p* > 0.05	F(1,36) = 2.15, *p* > 0.05	F(1,36) = 1.79, *p* > 0.05
Hypothalamus	F(1,33) < 1	F(1,33) < 1	F(1,33) = 4.31 *
*Htr4*			
Hippocampus	F(1,35) < 1	F(1,35) < 1	F(1,35) = 8.42 **
Midbrain	F(1,34) = 1.65, *p* > 0.05	F(1,34) < 1	F(1,34) < 1
Frontal cortex	F(1,36) < 1	F(1,36) = 1.02, *p* > 0.05	F(1,36) < 1
Hypothalamus	F(1,33) < 1	F(1,33) < 1	F(1,33) = 7.46 *
*Htr7*			
Hippocampus	F(1,36) = 13.46 ***	F(1,36) < 1	F(1,36) < 1
Midbrain	F(1,34) < 1	F(1,34) < 1	F(1,34) < 1
Frontal cortex	F(1,36) = 16.44 ***	F(1,36) = 1.14, *p* > 0.05	F(1,36) < 1
Hypothalamus	F(1,33) = 3.43, *p* = 0.073	F(1,33) < 1	F(1,33) = 6.40 *
*Tph2*			
Midbrain	F(1,34) < 1	F(1,34) = 1.02, *p* > 0.05	F(1,34) = 5.16 *
*Slc6a4*			
Midbrain	F(1,34) < 1	F(1,34) < 1	F(1,34) < 1

Genotype: C57Bl/6 (BL/6) or CBA/Lac (CBA). Photoperiod: standard (14L10D) day or short (4L20D) day. Interaction: genotype × photoperiod. * *p* < 0.05; ** *p* < 0.01; *** *p* < 0.001 (significant values are shown in bold).

**Table 3:** ANOVA results for the effects of genotype, photoperiod, and their interaction on the mRNA levels of BDNF-related genes in the different brain areas of catalepsy-resistant C57Bl/6 and catalepsy-prone CBA/Lac mice.

	Effect of Genotype	Effect of Photoperiod	Effect of Interaction
*Bdnf*			
Hippocampus	F(1,36) = 31.71 ***	F(1,36) = 1.93, *p* > 0.05	F(1,36) < 1
Midbrain	F(1,35) = 2.83, *p* > 0.05	F(1,35) < 1	F(1,35) < 1
Frontal cortex	F(1,36) = 2.63, *p* > 0.05	F(1,36) = 11.73 **	F(1,36) = 6.60 *
Hypothalamus	F(1,33) < 1	F(1,33) < 1	F(1,33) = 7.29 *
*Ntrk2*			
Hippocampus	F(1,36) = 6.73 **	F(1,36) = 4.63 *	F(1,36) = 1.48, *p* > 0.05
Midbrain	F(1,35) < 1	F(1,35)= 4.44 *	F(1,35) < 1
Frontal cortex	F(1,35) < 1	F(1,35) = 6.38 *	F(1,35) = 1.87, *p* > 0.05
Hypothalamus	F(1,33) = 7.29 *	F(1,33) < 1	F(1,33) = 5.63 *
*Ngfr*			
Hippocampus	F(1,36) < 1	F(1,36) = 4.51 *	F(1,36) < 1
Midbrain	F(1,35) = 8.02 **	F(1,35) < 1	F(1,35) = 4.86 *
Frontal cortex	F(1,36) < 1	F(1,36) < 1	F(1,36) = 2.04, *p* > 0.05
Hypothalamus	F(1,33) = 1.43, *p* > 0.05	F(1,33) < 1	F(1,33) < 1
*Creb1*			
Hippocampus	F(1,36) = 94.78 ***	F(1,36) = 2.52, *p* > 0.05	F(1,36) < 1
Midbrain	F(1,35) < 1	F(1,35) = 1.72, *p* > 0.05	F(1,35) < 1
Frontal cortex	F(1,35) = 21.98 ***	F(1,35) < 1	F(1,35) < 1
Hypothalamus	F(1,33) = 4.77 *	F(1,33) = 1.55, *p* > 0.05	F(1,33) = 16.81 ***

Genotype: C57Bl/6 (BL/6) or CBA/Lac (CBA). Photoperiod: standard (14L10D) day or short (4L20D) day. Interaction: genotype × photoperiod. * *p* < 0.05; ** *p* < 0.01; *** *p* < 0.001 (significant values are shown in bold).

**Table 4:** Summary of differences in the short photoperiod effects between catalepsy-resistant C57Bl/6 (BL/6) and catalepsy-prone CBA/Lac (CBA) male mice.

	Index	Short Photoperiod Exposure	
	Body weight	BL/6 =	CBA ↑
Behavior	FST depressive-like immobility TST depressive-like immobility	BL/6 = BL/6 ↑	CBA ↑ CBA =
	Cataleptic immobility	No	CBA ↑
5-HT brain system: metabolism	5-HT level in Ht and in Hc 5-HIAA level in Ht	BL/6 =	CBA ↓
5-HT brain system: expression of key genes	*Htr1a* mRNA in Hc *Htr4* and *Htr7* mRNA in Ht *Tph2* mRNA in Mb	BL/6 =	CBA ↓
	*Htr1a mRNA* in FC *Htr4* mRNA in Hc	BL/6 ↓	CBA =
Expression of BDNF-related genes	*Bdnf* mRNA in FC and Ht *Ntrk2* mRNA in FC	BL/6 =	CBA ↓
	*Ngfr* mRNA in Mb *Ntrk2* mRNA in Hc	BL/6 ↑	CBA =
	*Creb1* mRNA in Ht	BL/6 ↑	CBA ↓

↑ increase, ↓ decrease, = no effect in comparison with the standard photoperiod group of the same strain. Mb—midbrain, Hc—hippocampus, FC—frontal cortex, Ht—hypothalamus; 5-HT—serotonin, 5-HIAA—5-hydroxyindoleacetic acid, *Htr1a*—5-HT1A receptor gene, *Htr4*—5-HT4 receptor gene, *Htr7*—5-HT7 receptor, *Tph2*—tryptophan hydroxylase 2 gene, *Slc6a4*—serotonin transporter gene, *Bdnf*—brain-derived neurotrophic factor (BDNF), *Ntrk2*—neurotrophic receptor tyrosine kinase 2 gene (TrkB receptor gene), *Ngfr*—nerve growth factor receptor gene (p75 receptor), *Creb1*—CAMP responsive element binding protein 1 (CREB1).

**Table 5:** The primer sequences, annealing temperatures, and PCR product lengths.

Gene	Sequence	Annealing Temperature, °C	Product Length, bp
*Htr1a*	F 5′-ctgtgacctgtttatcgccctg-3′ R 5′-gtagtctatagggtcggtgattgc-3′	62	109
*Htr2a*	F 5′-agaagccaccttgtgtgtga-3′ R 5′-ttgctcattgctgatggact-3′	61	169
*Htr4*	F 5′-gtggtgtgtcttcatggtcaac-3′ R 5′-ctcctgcccgttgtaacatc-3′	62	155
*Htr7*	F 5′-ggctacacgatctactccaccg-3′ R 5′-cgcacactcttccacctccttc-3′	65	198
*Tph2*	F 5′-cattcctcgcacaattccagtcg-3′ R 5′-cttgacatattcaactagacgctc-3′	61	239
*Scl6a4*	F 5′-cgctctactacctcatctcctcc-3′ R 5′-gtcctgggcgaagtagttgg-3′	63	101
*Bdnf*	F 5′- tagcaaaaagagaattggctg-3′ R 5′- tttcaggtcatggatatgtcc-3′	59	255
*Ntrk2*	F 5′-cattcactgtgagaggcaacc-3′ R 5′-atcagggtgtagtctccgttatt-3′	63	175
*Ngfr*	F 5′-acaacacccagcacccagga-3′ R 5′-cacaaccacagcagccaaga-3′	62	171
*Creb1*	F 5′-gctggctaacaatggtacggat-3′ R 5′-tggttgctgggcactagaat-3′	64	140
*Polr2a*	F 5′-tgtgacaactccatacaatgc-3′ R 5′-ctctcttagtgaatttgcgtact-3′	61	194

*Htr1a*—5-HT1A receptor gene, *Htr2a*—5-HT2A receptor gene, *Htr4*—5-HT4 receptor gene, *Htr7*—5-HT7 receptor, *Tph2*—tryptophan hydroxylase 2 gene, *Slc6a4*—serotonin transporter gene, *Bdnf*—brain-derived neurotrophic factor (BDNF), *Ntrk2*—neurotrophic receptor tyrosine kinase 2 gene (TrkB receptor gene), *Ngfr*—nerve growth factor receptor gene (p75 receptor), *Creb1*—CAMP responsive element binding protein 1 (CREB1), *Polr2a*—DNA-directed RNA polymerase II subunit RPB1.

## Data Availability

The datasets generated during the current study are available from the corresponding author on reasonable request.
